# A Theory of Challenge and Threat States in Athletes: A Revised Conceptualization

**DOI:** 10.3389/fpsyg.2020.00126

**Published:** 2020-02-06

**Authors:** Carla Meijen, Martin Turner, Marc V. Jones, David Sheffield, Paul McCarthy

**Affiliations:** ^1^Department of Sport and Exercise Science, Faculty of Sport, Health and Applied Science, St Mary’s University, Twickenham, London, United Kingdom; ^2^Department of Psychology, Faculty of Health, Psychology and Social Care, Manchester Metropolitan University, Manchester, United Kingdom; ^3^School of Human Sciences, College of Life and Natural Sciences, University of Derby, Derby, United Kingdom; ^4^Department of Psychology, School of Health and Life Sciences, Glasgow Caledonian University, Glasgow, United Kingdom

**Keywords:** stress, performance, motivation, emotions, biopsychosocial

## Abstract

The Theory of Challenge and Threat States in Athletes (TCTSA) provides a psychophysiological framework for how athletes anticipate motivated performance situations. The purpose of this review is to discuss how research has addressed the 15 predictions made by the TCTSA, to evaluate the mechanisms underpinning the TCTSA in light of the research that has emerged in the last 10 years, and to inform a revised TCTSA (TCTSA-R). There was support for many of the 15 predictions in the TCTSA, with two main areas for reflection identified: to understand the physiology of challenge and to re-evaluate the concept of resource appraisals. This re-evaluation informs the TCTSA-R, which elucidates the physiological changes, predispositions, and cognitive appraisals that mark challenge and threat states. First, the relative strength of the sympathetic nervous system response is outlined as a determinant of challenge and threat patterns of reactivity and we suggest that oxytocin and neuropeptide Y are also key indicators of an adaptive approach to motivated performance situations and can facilitate a challenge state. Second, although predispositions were acknowledged within the TCTSA, how these may influence challenge and threat states was not specified. In the TCTSA-R, it is proposed that one’s propensity to appraise stressors is a challenge that most strongly dictates acute cognitive appraisals. Third, in the TCTSA-R, a more parsimonious integration of Lazarusian ideas of cognitive appraisal and challenge and threat is proposed. Given that an athlete can make both challenge and threat primary appraisals and can have both high or low resources compared to perceived demands, a 2 × 2 bifurcation theory of challenge and threat is proposed. This reflects polychotomy of four states: high challenge, low challenge, low threat, and high threat. For example, in low threat, an athlete can evince a threat state but still perform well so long as they perceive high resources. Consequently, we propose suggestions for research concerning measurement tools and a reconsideration of resources to include social support. Finally, applied recommendations are made based on adjusting demands and enhancing resources.

## Introduction

Jessica^1^ is standing at the start of an important road race, with an undulating course, the pressure mounting and her heart beating in her throat, she knows that the race will be physically and mentally demanding. Jessica has trained hard for this. Jessica believes that she is capable of pacing herself and feels ready to tackle the hilly course. She strides off rhythmically, able to follow her pre-race plan, deal with unforeseen events, and achieve a personal best. In this example, we would consider that Jessica is in a challenge state. To Jessica’s left, Sarah stands at the start of the same race. Just like for Jessica, Sarah feels her heart rate increase, and she knows that the race will be demanding and has also trained hard. However, in contrast to Jessica, Sarah does not believe that she is capable of pacing herself and does not feel ready to tackle the hilly course. She strides off enthusiastically but cannot find her rhythm and is unable to follow her pre-race plan. She deals with unforeseen events poorly and gets distracted and completes the race outside of her expected time. In this example, we would consider that Sarah is in a threat state. These examples illustrate that despite both athletes entering a stressful situation, stress is not always harmful (Cox, 1978), and can in fact benefit performance (Jessica) and related well-being outcomes (see also Selye, 1956).

The idea that stress can be both adaptive and maladaptive for skilled athletic performance is at the core of the Theory of Challenge and Threat States in Athletes (TCTSA; Jones et al., 2009). The TCTSA offers a psychophysiological framework for how athletes anticipate motivated performance situations (i.e., personally relevant events), such as competitions or selection events, based on an athlete’s interpretation of the situational demands and their available resources. The TCTSA proposes that athletes can approach performance situations in either a challenge state or a threat state. In anticipation of a motivated performance situation, an athlete who has high self-efficacy, high perceived control, and an approach focus, is likely to experience a challenge state; on the other hand, if an athlete has low self-efficacy, low control, and an avoidance focus, they are likely to experience a threat state. The TCTSA draws on prominent transactional appraisal theories of stress and emotion, such as the biopsychosocial model (BPSM) of challenge and threat (Blascovich and Mendes, 2000), and the work of Lazarus and Folkman (1984) and Dienstbier (1989). In developing the TCTSA, Jones et al. (2009) aimed to describe the cognitive, affective, and physiological aspects of challenge and threat states along with potential performance consequences. In particular, in the TCTSA, a unique combination of psychological constructs interacts to determine challenge and threat states. A number of hypotheses are also put forth by Jones et al. including the assertions that high-intensity negative emotions can be experienced in a challenge state, but are perceived as facilitative for performance, and that challenge and threat states influence performance through effort, attention, decision-making, and physical functioning.

### Justification and Aims

Two recent reviews concerning challenge and threat states (Behnke and Kaczmarek, 2018; Hase et al., 2019) have focused on how challenge and threat states influence performance. But the TCTSA makes broader predictions about competitive anticipatory states that go beyond performance outcomes, and therefore, a review of the research that focuses on challenge and threat states in sport more broadly is warranted to help guide future research and practice. Furthermore, considering that the TCTSA was published 10 years ago, it is timely to review the research conducted within sport environments and propose refinements to the theory in order advance challenge and threat theory in sport settings. When proposing the TCTSA in 2009, Jones et al. focused on explaining why athletes may perceive an upcoming situation as a challenge or a threat, and what informs the perceived availability of resources in a sporting context. One of the primary aims at the time of proposing the theory was to guide applied work, and outline specific predictions that could be tested within a sporting performance context. The present review extends beyond that, and the aim is to re-evaluate the TCTSA, and in light of the evidence that has amassed since the 2009 publication of the TCTSA, to propose a revised theory (TCTSA-Revised[R]). In the TCTSA-R, we reconsider the cognitive appraisal network and provide a more detailed portrayal of how athletes can approach motivated performance situations adaptively, in a challenge state. Therefore, the aims of the current paper are fourfold. First, to provide an overview of how the research has addressed the 15 predictions made by the TCTSA. Second, to explain the mechanisms underpinning the TCTSA in light of the research that has emerged in the last 10 years. Third, the role of social support and well-being in challenge and threat states is considered. Finally, considering the initial predictions and emerging research we propose the TCTSA-R with guidance for future research and applied work.

## Overview of Theory of Challenge and Threat States in Athletes

In its original conception, there were four key components of the TCTSA: demand appraisals and motivational states, resource appraisals, physiological responses, and emotional consequences. First, building on the BPSM, for challenge and threat states to occur, the athlete must perceive the demands of a situation as dangerous (physical and or esteem), uncertain, and requiring of effort (physical and or mental). To clarify, a motivated performance situation, or motivational state, in a sporting context is often considered a situation in which there is pressure on the athlete to perform, and drawing on Lazarus’ work (Lazarus, 1999), is usually personally relevant to the athlete. Competitive sporting situations are typically motivational states because they are personally meaningful to the athlete, the outcome is usually unknown before the start (uncertainty), there is a potential for danger (ego could be at stake when an athlete is worried about the outcome), and effort is required to fulfill athletic potential.

Second, in the TCTSA, it is proposed that resource appraisals comprise three interrelated constructs, namely self-efficacy, perceptions of control, and achievement goals. Self-efficacy is one’s belief in their abilities to successfully accomplish a task (Bandura, 1997). Control is closely linked to self-efficacy and includes acceptance and awareness of factors that are within and outside an individual’s personal control (Jones et al., 2009). Achievement goals are closely linked to an individual’s motivation to participate in sport, and in the TCTSA are drawn from a 2 × 2 achievement goal framework that comprises mastery and performance achievement goals, aligned with either goal approach or goal avoidance (Elliot and McGregor, 2001). The TCTSA outlines that, typically, a challenge state is characterized by high levels of self-efficacy, a high perception of control, and a focus on approach goals, whereas a threat state is proposed to be characterized by low self-efficacy and control, and a focus on avoidance goals (Jones et al., 2009). In a challenge state, the perceived resources are sufficient to deal with the demands of the situation, whereas in a threat state the demands outweigh the perceived resources. There is an important distinction to make between the challenge and threat evaluation and Lazarus’ conceptualization. That is, in the original BPSM, and adapted by the TCTSA, challenge and threat states were considered to be the “end result” of the evaluation of demands and resources (Seery, 2011). This differs from Lazarus’ appraisal process where challenge and threat are considered to be a result of primary appraisals, where challenge reflects a potential for gain, and threat reflects a potential for harm. For Lazarus (1999), this primary appraisal is met with secondary appraisal in which coping potential is appraised. The BPSM and TCTSA deviate from the primary and secondary appraisals concepts in favor of demand and resource appraisals in their formulation of challenge and threat. This consideration is important as it informs the two distinct physiological responses that are associated to challenge and threat states whereby sufficient resources that outweigh demands correspond to distinct physiological responses that signify a challenge state. In contrast, insufficient resources that do not outweigh demands correspond to distinct physiological responses that signify a threat state (see Jones and Turner, 2014).

Borrowing from the biopsychosocial model of arousal regulation (Blascovich and Mendes, 2000), the TCTSA outlines that the two distinct physiological responses that mark challenge and threat states can be measured using cardiovascular (CV) reactivity patterns indicative of changes in the stress systems (Dienstbier, 1989; Blascovich, 2008). It was proposed that a challenge state is characterized by increased sympathetic-adreno-medullary (SAM) activity accompanied by an increase in catecholamine release, indexed by increased heart rate (HR) and cardiac output (CO), attenuated preejection period (PEP), and decreased total peripheral resistance (TPR). In essence, a challenge state promotes efficiency of energy (glucose) delivery, and use, due to increased blood flow to the brain and muscles, higher blood glucose levels (fuel for the nervous system), and an increase in free fatty acids that can be used by muscles as fuel (e.g., Dienstbier, 1989). Therefore, a challenge state facilitates improved decision-making, effective and maintained cognitive function, decreased likelihood of reinvestment, efficient self-regulation, and increased anaerobic power, all of which are likely to lead to successful sports performance (Jones et al., 2009). In a threat state, it was proposed that increased SAM activity is accompanied by increased pituitary-adreno-cortical (PAC) activity, and subsequent cortisol release. Thus, increased HR and attenuated PEP occurs, but with an increase or stabilization in TPR, and a small increase or stabilization in CO. Thus, in a threat state SAM activity is tempered and therefore efficiency of energy use does not occur as blood flow to the brain and muscles is not increased and the mobilization of usable energy is slower than in a challenge state (e.g., Dienstbier, 1989). Therefore, a threat state leads to ineffective decision-making and cognitive function, increased likelihood of reinvestment, inefficient self-regulation, and decreased anaerobic power (compared to a challenge state), all of which are likely to lead to unsuccessful sports performance (Jones et al., 2009). In short, in a challenge state, SAM activation is fast-acting and represents the mobilization of energy for action (fight or flight) and coping. A threat state accompanies slow-acting PAC (and SAM) activation and represents a “distress system” associated with perceptions of actual harm (Blascovich and Tomaka, 1996).

Finally, the TCTSA also outlined the emotional consequences related to challenge and threat states. In particular, it was suggested that positive emotions are *typically* associated with a challenge state, and negative emotions with a threat state. This is, however, influenced by how facilitative or debilitative a person perceives their emotional state to be, in line with Jones’ (1995) model of debilitative and facilitative competitive state anxiety. That is, an athlete can experience anxiety before a competition, but can perceive this anxiety to be facilitative for their performance. Together, challenge and threat states can influence performance through decision-making, cognitive functioning, task engagement, and physical functioning. Typically, it is suggested that a challenge state is beneficial for athletic performance (Jones et al., 2009).

## Review of Research of Challenge and Threat States in Sport

Since proposing the TCTSA in 2009, the theory has been referenced across a range of domains besides sport. For example, the TCTSA has been considered in aviation (Vine et al., 2015), surgery (Moore et al., 2014), sport fans behavior (Sanderson, 2016), change management in business (Slater et al., 2016), public speaking tasks (Trotman et al., 2018), and visual search tasks (Frings et al., 2014; Laborde et al., 2015). In addition, Turner and Barker (2014a) produced a detailed application of the TCTSA in business settings, in which “performance” was considered to be broader than athletic skill execution. Considering that the original focus of the TCTSA was how athletes approach competitive sporting situations, we will only discuss studies that have focused on challenge and threat states in sport settings and or sports-related tasks. In the next section, the key findings of studies that have cited the TCTSA and appeared to have tested one or more of the 15 predictions of the TCTSA will be summarized.

From the sport-related studies that have cited the TCTSA, or measured challenge and threat states in a sporting context but did not cite the TCTSA, a minority of studies have measured cardiovascular responses. Fine motor skills tasks such as golf putting (Freeman and Rees, 2009; Moore et al., 2013a,b; Kingsbury et al., 2014), dart throwing (Moore et al., 2018), virtual ball task (Huber et al., 2016), carom billiard (Di Corrado et al., 2015), and shooting (Rossato et al., 2018) were used in the majority of the studies that measured performance as an outcome. Other researchers assessed performance using cricket batting performance (Turner et al., 2013) or soccer match performance (Dixon et al., 2019). Some studies used speech tasks to assess challenge and threat states (Allen et al., 2012; Meijen et al., 2014) in a sport sample, whereas other studies employed reflective diaries to ask athletes about their challenge and threat experiences (e.g., Nicholls et al., 2012) or interviews and observations (Massey et al., 2013; Didymus and Fletcher, 2017).

### The Predictions of the Theory of Challenge and Threat States in Athletes: What Do We Know Now?

When the TCTSA was published, 15 predictions were proposed (see Table 1). Typically, in support of prediction 1, studies where cardiovascular responses were measured found that demand appraisals led to an increase in heart rate. In the majority of the studies, danger, uncertainty, and effort were manipulated as part of the research design. For example, participants would be asked to perform in front of assessors (Turner et al., 2012; study 2), were told that they would be compared to others (Moore et al., 2012, 2013b; Turner et al., 2012; Mosley et al., 2017; Sammy et al., 2017; Brimmell et al., 2019), that they would be interviewed if they performed poorly (Moore et al., 2012, 2013b; Brimmell et al., 2019), that they would be judged by coaches (Turner et al., 2013), and/or that they would be videotaped (Moore et al., 2012; Turner et al., 2012; Mosley et al., 2017; Brimmell et al., 2019).

**Table 1:** TCTSA: An overview of the original predictions made (adapted from Jones et al., 2009).

	Prediction	Supported/Partially supported/Mixed support/ Not tested
1	Demand appraisals reflect the perception and assessment of danger, uncertainty, and effort required in a situation and is reflected by increase in HR	Supported
2	A challenge state is experienced when an athlete’s resource appraisals include high self-efficacy, high perceptions of control, and approach goals	Mixed support
3	A threat state is experienced when an athlete’s resource appraisals include low self-efficacy, low perceived control, and avoidance goals	Mixed support
4	Increased SAM activation and the release of epinephrine and norepinephrine as measured by increased cardiac activity and decreased TPR reflects a challenge response	Not tested
5	Increased SAM and PAC activation and the release of cortisol as measured by increased cardiac activity and either no change or increased TPR reflects a threat response	Not tested
6	A challenge state is typically associated with positively valenced emotions	Partially supported
7	A threat state is typically associated with negatively valenced emotions	Partially supported
8	Emotions experienced in a challenge state are perceived as facilitative to performance	Supported
9	Emotions experienced in a threat state are perceived as debilitative to performance	Supported
10	Athletes in a challenge state have greater self-regulatory resources available for the task demands because of a need for less self-regulation	Partially supported
11	The efficiency and effectiveness of cognitive functioning is lower in a threat state because of anxiety	Partially supported
12	Anxiety experienced in a threat state will not lead to reinvestment	Partially supported
13	There is less engagement when an athlete is in a threat states because of the use of avoidance strategies	Not tested
14	Decision-making will be facilitated in a challenge state	Partially supported
15	Anaerobic power will be positively impacted in a challenge state	Partially supported

A majority of the studies appeared to test predictions 2 and 3, examining the associations between self-efficacy, perceptions of control, and achievement goals, using self-report measures or interviews (for example, Howle and Eklund, 2013; Meijen et al., 2013). Meijen et al. (2013) found that avoidance goals were positively related to a threat perception, and approach goals and self-efficacy negatively predicted a threat perception. We also identified that a substantial number of studies explored the relationship between challenge and threat states and emotional responses (predictions 6 and 7). Typically, these studies identified a positive relationship between anxiety and threat states (for example, Williams et al., 2010). Overall, there is mixed evidence to support the proposed relationships between the resource appraisals (self-efficacy, perceptions of control, achievement goals), cardiovascular indices of challenge and threat, and emotions. Some published studies support the proposed relationships (Trotman et al., 2018), whereas others do not (Turner et al., 2012, 2013; Dixon et al., 2019). Indeed, in one study, higher levels of self-efficacy were associated with a threat state, which is contrary to the TCTSA (Meijen et al., 2014). Moreover, Dixon et al. (2019) showed that challenge CV reactivity positively predicted future soccer performance (rated by players and coaches), but that athletes with a blunted CV response performed worse than challenge and threat responders and that there was a weak association between self-report data and cardiovascular responses. Interestingly, the findings of Trotman et al. (2018) show support for the central tenets of the TCTSA during competitive stress, but not social stress. This suggests that the type of task may have an impact on the relationship between resource appraisals and cardiovascular reactivity, and that blunted cardiovascular responses need to be considered (see also Wormwood et al., 2019). Moreover, whereas there is mixed evidence for the link between resource appraisals and physiological responses, there is more consistent evidence that improving resource appraisals benefits a challenge state (e.g., Turner et al., 2014).

The TCTSA further predicted (predictions 4 and 5), in line with the BPS model of arousal regulation, that an increase in SAM activation alone as indicated by increased epinephrine and norepinephrine reflects a challenge state. Increased SAM activation combined with PAC activation was suggested to characterize a threat state. No research has assessed the underlying neuroendocrine responses, rather most studies used the challenge and threat index (based on Blascovich et al., 2004) to assess the challenge and threat cardiovascular response (Allen et al., 2012; Moore et al., 2012, 2013b; Turner et al., 2012, 2013, 2014; Vine et al., 2013; Meijen et al., 2014; Sammy et al., 2017) to differentiate between challenge and threat states. This challenge and threat index is calculated by converting the CO and TPR reactivity scores into Z scores and summing them, with CO being assigned a weight of +1 and TPR a weight of −1. High scores indicate a challenge, and low scores a threat. Some of these studies also reported cardiac output and total peripheral reactivity scores separately (i.e., Turner et al., 2012; Meijen et al., 2014). Although most of the studies identified distinct challenge and threat cardiovascular reactivity patterns (Moore et al., 2012; Turner et al., 2014; Sammy et al., 2017), some studies failed to observe a distinct cardiovascular reactivity pattern (Meijen et al., 2014), and no studies have measured the underlying neuroendocrine responses.

The interpretation of emotional states (prediction 8 and 9) was typically assessed by experimental studies focused on reappraising of arousal (Moore et al., 2015; Sammy et al., 2017). Together they found that reappraising arousal had the potential to promote a challenge state. Furthermore, Williams et al. (2010) used imagery to manipulate challenge and threat states and found that participants interpreted anxiety as more facilitative during the challenge script.

The prediction that there is a need for less self-regulation in a challenge state was predominantly tested in relation to use of coping strategies (Allen et al., 2012; Mosley et al., 2017) (prediction 10). Some support was evident for this prediction, in particular those who responded to a situation as a threat seemed to draw on more problem-oriented and emotion-focused coping (Allen et al., 2012). Furthermore, the presence of a pacer, as a coping strategy, can reduce the required sources and subsequently lead to less need for self-regulation (H. Jones et al., 2016).

Predictions 11 and 12 outline that anxiety decreases the efficiency and effectiveness of cognitive functioning in a threat state (prediction 11), but that in a challenge state anxiety does not lead to reinvestment (prediction 12). Some support was provided for these predictions, Sammy et al. (2017) found that performance did not improve more after arousal reappraisal (which was suggested to promote a challenge state) compared to a control group. They suggested that, in line with attentional control theory (Eysenck et al., 2007), participants may have used compensatory strategies such as increased effort to deal with the pressure from the task. Furthermore, after a challenge manipulation, experienced golfers used less conscious processing (Moore et al., 2013b). Although Robazza et al. (2018) did not measure cardiovascular reactivity patterns, they did suggest that, for junior orienteers, a worsened psychobiological state (similar to a threat state) together with reduced “top-down executive functions” seemed to negatively affect performance.

Prediction 13 states that athletes engage less in competition when they are in a threat state. That is, athletes draw more on avoidance strategies, and may engage in freezing where they may perceive a demand to be dangerous and therefore disengage themselves from the situation (Jones et al., 2009). In practical terms, this may be an athlete who decides to avoid going into a tackle at a rugby match. Although there were no experimental studies focusing on this prediction, Howle and Eklund (2013) found that a challenge state was associated with lower avoidance goals.

Prediction 14 of the TCTSA states that being in a challenge state can have a positive influence on decision-making. In one study, there was a positive relationship between threat appraisals and autocratic coaching behaviors (Dixon et al., 2017). In addition, although not conducted with an athletic sample, Turner et al. (2012) found that a challenge CV state was related to superior accuracy on the Stroop Test, used to assess decision-making.

Only one study (Wood et al., 2018b) has directly considered the impact of challenge states on anaerobic power (prediction 15). In this study, there was a relationship between challenge appraisals and anaerobic power in a cycling task, with challenge appraisals being associated with greater anaerobic power, however, there was no relationship between cardiovascular reactivity and anaerobic power in a cycling task. It was noted by the authors (Wood et al., 2018b) that methodological issues, such as the length of time between baseline trials and performance impacted power levels during the test itself and therefore is a need for more research on this prediction. The limited research may not be surprising considering the physiological changes that the body undergoes from rest to vigorous physical activity. The influence of experiencing a challenge state, however, could impact the perceived effort ratings of athletes (Jones et al., 2016).

Consideration of the sports-related studies that cited the TCTSA or measured challenge and threat states in a sporting context illustrates two main areas for reflection. The first is understanding the physiology of challenge and threat. That is, what are the physiological changes under stress that are reflected in the distinct patterns of cardiovascular reactivity and are there other physiological correlates or determinants of challenge and threat states? The second consideration is that the resource appraisals outlined in the TCTSA need re-evaluating as these do not consistently link to the proposed patterns of CV reactivity. Some of these findings may represent the social desirability inherent in self-report measures (cf. Meijen et al., 2014) or that the tasks used may not approximate sufficiently to competitive situations (cf. Trotman et al., 2018). Nevertheless, the inconsistent findings do require a second look, if not a re-evaluation, and reflection on whether other concepts, such as perceived social support, need to be considered as part of resource appraisals to better represent the social environment inherent to challenge and threat states.

### The Physiology of Challenge and Threat States

The physiological mechanisms underpinning and reflecting a challenge response in athletes was outlined in the BPSM and adapted by the TCTSA. In this section, we review the proposals in the TCTSA in more depth and we consider wider physiological markers which underpin, and reflect, challenge and threat states. Based on the work of Blascovich and Tomaka (1996) and Blascovich and Mendes (2000), it was proposed that a challenge state is characterized by activation of the sympathetic nervous system and accompanying increases in epinephrine and norepinephrine, evidenced by an increase in cardiac activity along with a decrease in peripheral vascular resistance. In contrast, a threat state is characterized not only by activity of the sympathetic nervous system, but also increased activity of the hypothalamic–pituitary–adrenal (HPA) axis, accompanying increases in cortisol, smaller increases in cardiac activity, and either no change or an increase in peripheral vascular resistance.

More recent explanations of the physiological underpinnings of challenge and threat states have focused on the temporal aspects of the sympathetic nervous system (SNS) response, where it was proposed that challenge states result from a quick SNS response which quickly habituates, whereas threat states have a slower rise in SNS activity which tends to stay elevated for a longer time (Epel et al., 2018). It is this response that is reflected in the differing patterns of challenge and threat cardiovascular reactivity. This explanation would fit within the timescales typically used in cardiovascular reactivity research, but again the mechanisms need further elucidating. Specifically, the release of norepinephrine under acute stress leads to vasoconstriction (Carter and Goldstein, 2015). Indeed, one criticism is that SAM activity is associated with the release of norepinephrine which has vasoconstrictive effects and, so, even if the release of epinephrine did reduce resistance through dilation, any effect could be offset by norepinephrine (Wright and Kirby, 2003). To explain the observed vasodilation, we propose that under conditions of challenge, SNS activation quickly dissipates (cf. Epel et al., 2018) and it is the decrease in sympathetic stimulation that allows *relative* vasodilation in the arterioles, reflected in decreased vascular resistance. Under conditions of threat, because the SNS activation does not dissipate, this is reflected in continued vasoconstriction (Webb, 2003). This is a testable hypothesis, best examined through manipulating challenge and threat states, although to the best of our knowledge has not been explored. Specifically, minute-by-minute analyses of individuals displaying challenge and threat cardiovascular reactivity should demonstrate for both groups an increase in vasoconstriction in the immediate seconds after the acute stressor (e.g., 60 s). Thereafter, the patterns should, however, diverge. Specifically, those who are challenged should show relative vasodilation indicating the absence of sympathetic stimulation, whereas those who are threatened should continue over the next few seconds (e.g., up to 120 s) to show vasoconstriction resulting from continued sympathetic stimulation.

After the initial few minutes of SNS response to the motivated performance setting, there may be further divergence of those exhibiting a challenge and threat response with greater levels of cortisol in those who are threatened. The arousal from HPA activation, which is greater in a threat state, will not dissipate quickly because cortisol has a much longer half-life (30–90 min; Kirschbaum and Hellhammer, 1994). In contrast, peak catecholamine (epinephrine, norepinephrine) responses should decline only to the level needed to sustain active coping (Dienstbier, 1989) and this may vary depending on the nature and demand of the sport. This is of course a difficult task considering challenge and threat states in athletes given different sports have different demands, and the feasibility of measuring physiological responses immediately before or during sporting performance may not be possible. What this also underlines is that, because the consequences of HPA axis activation are active for that amount of time, there is a stronger link with anticipatory appraisals than retrospective appraisals related to stress (Gaab et al., 2005). Whereas the explanation of challenge and threat states has focused on SNS and HPA activation, the parasympathetic nervous system may also play a role as outlined in this issue with potentially a withdrawal of the parasympathetic system being an indicator of a threat state (see Uphill et al., 2019 for a detailed discussion).

Considering the relevance of anticipatory appraisals for HPA axis activation, this links in well with our second consideration when reflecting on the TCTSA research. The TCTSA outlined specific resource appraisals that inform anticipatory appraisals; the research findings are, however, less consistent with the predictions. One of the potential limitations of how resource appraisals were set out in the TCTSA is that they were focused on individual resources to the neglect of social ones. Social support, however, was a component of resource appraisals described by Lazarus and Folkman (1984), and the importance of social environments in determining cardiovascular reactivity and performance has long been recognized (Carroll and Sheffield, 1998; Uchino et al., 2011). This consideration is relevant, as aspects such as perceived social support can influence anticipatory appraisals and anticipatory BP and hemodynamic responses to mental stress (Gramer and Reitbauer, 2010). To elaborate, although the TCTSA borrows from the biopsychosocial model of arousal regulation (Blascovich and Mendes, 2000), the TCTSA did not make specific predictions about the role of perceived social support. In addition, Dixon et al. (2017) found that coaches who appraised a stressor as a challenge were more likely to provide social support to their athletes. We propose that both the perception and provision of social support play an important part as a resource in anticipation of a motivated performance setting (Kirsch and Lehman, 2015), which can influence oxytocin levels (Heinrichs et al., 2003). Therefore, we will now focus on a brief overview of perceived social support, and how we see if fit in relation to challenge and threat states.

### Social Support in Challenge and Threat Research

Social support involves “an exchange of resources between at least two individuals perceived by the provider or recipient to be intended to enhance the well-being of the recipient” (Shumaker and Brownell, 1984, p. 13). It benefits self-confidence (Freeman and Rees, 2010), motivation, performance (Freeman and Rees, 2009; Tamminen et al., 2019), well-being (DeFreese and Smith, 2014), group cohesion, performance slumps and injury recovery (Madden et al., 1989; Udry, 1996) and competitive and personal stressors (Crocker, 1992; Rees and Hardy, 2000) as a situational characteristic implicit in the competitive stress process.

Though social support includes functional (i.e., support exchanges), structural (i.e., support network), and perceptual (i.e., support appraisal) aspects (Bianco and Eklund, 2001), sport researchers focused upon functional aspects (Arnold et al., 2018) and perceived availability of support and support received (Freeman and Rees, 2010). Perceived support comprises four dimensions (i.e., emotional, esteem, informational, and tangible) and matters more to outcome variables such as performance and self-confidence than support actually received.

Research shows that social support influences outcomes directly (i.e., main effects model) or indirectly (i.e., stress buffering hypothesis). In the main effects model, researchers identified the association between social support and performance factors in tennis (Rees et al., 1996; Rees and Hardy, 2004) and performance outcomes in golf (Rees et al., 2007; Rees and Freeman, 2009). According to the stress buffering hypothesis, social support can moderate the effects of stressors on outcomes. Perceived social support aids the appraisal process by redefining the situational threat and augmenting the individual’s perceived control and ability to cope. Together, such resources increase coping behaviors, self-efficacy with concomitant changes in the affective, physiological, and behavioral response to stress (Cohen et al., 2000; Rees and Hardy, 2004; Freeman and Rees, 2009, 2010; Rees and Freeman, 2009; Arnold et al., 2018).

The collected research holds that social support benefits psychological well-being and sport performance though researchers sometimes overlook the social constituent of the biopsychosocial trinity in the BPSM. Blascovich (2008) proposed social support to influence demand and/or resource evaluations; however, previous research examining the effect of perceived social support on cardiovascular reactivity to stress offered equivocal results (see Closa León et al., 2007; O’Donovan and Hughes, 2008). Moore et al. (2014) reported that perceptions of support availability had no significant influence on participants’ demand/resources evaluations, cardiovascular responses, or performance in a laparoscopic surgery task.

Perceived social support helps the athlete in motivated performance situations. Although self-relevant goals like a monetary reward might be important, one’s basic need to form and maintain social bonds (e.g., Baumeister and Leary, 1995) means that making a good impression (e.g., on the experimenter) might be a typical source of motivated performance in a laboratory setting (Seery, 2013). In ecologically diverse settings, the presence of others (e.g., social anxiety, social comparison, social power) primes a psychological response that could be mediated by the perceived social support of teammates, coaches, family, and friends, allowing athletes to locate resources to marshal the stressors encountered in motivation performance situations. Dixon et al. (2017) explored the relationships between challenge and threat cognitive appraisals and coaching behaviors in football coaches. Their results suggested that coaches with a tendency to appraise a stressor as a challenge are more likely to offer social support to their athletes. A series of stress reappraisal interventions (Jamieson et al., 2010, 2013) demonstrated better performance outcomes and diminished stress responses in participants who received the reappraisal instructions.

Clearly, psychosocial factors such as perceived social support can influence the cognitive appraisal process. Not only can perceived social support provide a stress buffer; Slater et al. (2016) propose that social support could also influence the perception of demand and resource appraisals. For example, an athlete who perceived high availability of social support may reasonably appraise less required effort due to shared problem solving, and less danger to esteem through the knowledge that no matter what happens (e.g., failure) they will be safe in their social group. For the resources, research has demonstrated how instructional sets that promote perceptions of high resources can lead to a challenge state (Turner et al., 2014), and this has clear ramifications for social support, particularly informational support. In anticipation of a competition, a number of people surrounding an athlete can provide information that could increase (and of course decrease) the athlete’s perceptions of self-efficacy, control, and goal orientation. A coach could encourage the athlete to reflect on successful performances in the past (self-efficacy); a teammate could orient the athlete toward aspects of the performance that they can control such as sticking to the game plan, or preparing in the right way (control); a friend could encourage the athlete to focus on the opportunity they have to demonstrate their many skills and abilities (approach goals). The role of the coach in athlete challenge and threat states is potentially important. Research (Slater et al., 2018) indicates that performers who perceive high connectedness (high relational identification) with a task leader report greater resource appraisals and performed better (in a cognitive task). Slater et al. also found that being led by an individual with whom participants felt low connectedness (low relational identification) elicited threat CV reactivity to a pressurized task (Study 3). It is important that athletes perceive that these support options are available, from people with whom they share a strong connection, and that they seek to use these opportunities for social support in anticipation of a motivated performance situation.

## Revising the Theory of Challenge and Threat States in Athletes

Thus far, we have set out the initial predictions of the TCTSA, reviewed research that has directly or indirectly tested predictions that were proposed when introducing the TCTSA; critically reviewed the physiological aspects and resources; and explained the relevance of adding perceived social support to the TCTSA as a resource appraisal. The story is complex, and with the TCTSA-R, we are cautious not to oversimplify the complexity of the human anticipatory responses that are at the core of the TCTSA. Nevertheless, we endeavor to clarify aspects of the TCTSA and make updated suggestions that we hope will stimulate debate and further (applied) research in relation to stress and athletic performance. The focus points of the TCTSA-R are: physiological changes, predispositions, and cognitive appraisal.

### Physiological Changes

The relative patterns of norepinephrine, epinephrine, and cortisol reflect responses to an acute stressor and underlying appraisals and are manifested in specific patterns of cardiovascular reactivity as outlined in the BPSM. The explanation that cardiovascular (CV) predictions derive from SAM and HPA activation has, however, been debated (Blascovich et al., 2003; Wright and Kirby, 2003). One criticism is that HPA axis activity is not sufficiently quick to be reflected in immediate CV reactivity. Indeed, the methodologies used to identify patterns of cardiovascular reactivity indicative of challenge and threat states show changes in a few minutes from baseline. Typically, studies have assessed and accordingly found challenge and threat states in the first minute (e.g., Blascovich et al., 2004; Moore et al., 2012; Meijen et al., 2014), 2 min (e.g., Blascovich et al., 2004; Allen et al., 2012), 3 min (e.g., Turner et al., 2012, 2013, 2014, study 2), or 4 min (e.g., Turner et al., 2014, study 1) following the onset of the stressors. This time frame is likely too short for CV reactivity to be influenced by HPA axis activity (Herman et al., 2016). Of course, this does not mean that HPA axis activity is not important in underpinning challenge and threat states, and HPA axis activity may differ across challenge and threat states. Rather, it means that the CV reactivity observed in the overwhelming majority of studies in which challenge and threat have been explored is not likely to have been influenced by HPA activity. In our revised TCTSA-R, we propose that oxytocin and neuropeptide Y are also both key indicators of an adaptive approach to motivated performance situations and differing levels can be reflected in challenge and threat states.

Neuropeptide Y (NPY) is a 36-amino acid peptide, and receptors for NPY are associated with three key locations in the brain that deal with stress: the amygdala, the hippocampus, and the locus coeruleus (Nulk et al., 2011). An increased level of NPY in the amygdala is associated with decreased feelings of anxiety, and increased levels generally may decrease the rate of locus coeruleus firing, resulting in lower levels of NE in the brain (Nulk et al., 2011). These propositions are supported by research in performance environments. Under acute stress, increases in norepinephrine and cortisol were significantly and positively associated with increases in plasma levels of NPY in military personnel, including Special Forces personnel in the US (Morgan III et al., 2000, 2001, 2002). The data from Morgan and colleagues suggest that levels of NPY are significantly and negatively associated with the subjective reports of stress. NPY has a counterbalancing effect to corticotropin-releasing hormone (CRH) and the balance between these two biochemicals is key, with CRH needed to maintain the stress response, while NPY is needed to counteract long-term damage caused by prolonged stress (Nulk et al., 2011). It was also suggested by Morgan and colleagues that a rise in peripheral plasma NPY (which was what was assessed in the military studies by Morgan and colleagues) may in itself exert central effects as peripheral infusion of NPY has been showing to have a central effect of decreasing HPA axis activation (cf. Antonijevic et al., 2000). In short, NPY seems to moderate the stress response allowing a helpful, rather than unhelpful, stress response.

A second biochemical that may play this role of moderating the stress response is oxytocin. Oxytocin is a neuropeptide produced in the hypothalamus that plays an important role in prosocial behaviors (Heinrichs et al., 2003). There is consistent evidence that oxytocin is associated with lower levels of cortisol under acute stress (e.g., Ditzen et al., 2009; Linnen et al., 2012; Cardoso et al., 2013; Robyn et al., 2016). The dampening effect of oxytocin on cortisol may, however, only occur in tasks that are sufficiently stressful to elicit a strong HPA axis response (Cardoso et al., 2014). This is important in athletic samples because oxytocin rises in response to perceived social support (e.g., Kubzansky et al., 2012; Robyn et al., 2016) and so the provision of support by significant others, coaches, team-mates, and audiences may be an important factor in facilitating challenge states (Turner and Barker, 2014b). Indeed, there is evidence that under a stressful speech and mathematics task, participants who were given oxytocin, compared to placebo participants, exhibited a trend (albeit non-significant) toward greater increases in CO indicating greater SNS activation in those with higher levels of oxytocin (Kubzansky et al., 2012). The mechanism by which oxytocin would impact SNS activation does need elucidating; however, there does seem preliminary evidence at least, certainly around HPA activation, that oxytocin may be an important factor in determining a challenge response.

### Predispositions

At its inception, it was acknowledged within the TCTSA that predisposition aspects including perfectionism, optimism, and hardiness influence challenge and threat states. We did, however, not specify the direction of how these dispositional factors influence challenge and threat states as our intention was to focus on the dynamicity of the state responses. In the revised theory, we provide some greater clarity on how dispositional style relates to challenge and threat states.

The notion that predispositions are an important part of cognitive appraisal is not new. In his early works, Lazarus recognized that the extent to which a situation is appraised as stressful or not can be influenced by dispositions (e.g., disposition to deny threat; Speisman et al., 1964). There is a vast array of predispositional factors that could influence cognitive appraisals ranging from genetics, to personality characteristics. A more promising predisposition that is nested within challenge and threat theory is the notion of trait challenge and threat. Contemporary research with elite rowers (Cumming et al., 2017) shows that predisposed cognitive appraisal style is associated with, and further predicts, subsequent state cognitive appraisals. Specifically, predisposed challenge was associated with event-specific state challenge, and predisposed threat was associated with event-specific state threat, on approach to subsequent motivated performance situations. This evidence from elite sport supports previous research (Skinner and Brewer, 2002) that also found that predisposed cognitive appraisal style can predict subsequent cognitive appraisals. There is also some evidence that irrational beliefs, as proposed with rational emotive behavior therapy (REBT), form an important part of the cognitive appraisal network (e.g., David et al., 2002), and that higher irrational beliefs are related to greater threat (Dixon et al., 2017; Evans et al., 2018). For example, in a recent study in this issue, golfers approaching a motivated performance situation with high irrational beliefs were more likely to evaluate the upcoming competition as a threat (Chadha et al., 2019). In line with TCTSA postulations, greater threat was related to greater negative emotion, greater competitive anxiety, and a less facilitative interpretation of anxiety. Irrational beliefs are considered to be “deep” cognitions akin to schemas or core beliefs, which are considered to be trait-like or dispositional (Turner, 2016). Thus, we argue that although a complex constellation of predispositional factors could influence acute cognitive appraisal, it is perhaps one’s propensity to hold irrational core beliefs and one’s proclivity to appraise stressors as a challenge that most powerfully dictate acute cognitive appraisals.

### Cognitive Appraisal

Cognitive appraisal in the TCTSA deviates from Lazarusian notions of cognitive appraisal in three important ways. First, whereas the BPSM and the TCTSA express the importance of demand and resource appraisals in challenge and threat states, Lazarus’ cognitive appraisal theory suggests that challenge and threat emerge from primary appraisals of motivational relevance, and goal congruence. Second, the TCTSA does not consider reappraisal in its network of psychophysiological responses. It is possible to reappraise situations that have already been subject to cognitive appraisal (see Gross, 1998, for review). In other words, that which was once appraised as a threat can be reappraised as a challenge, and vice versa. Third, in the TCTSA, challenge and threat are the result of cognitive appraisal, but for Lazarus (1999), challenge and threat are a part of cognitive appraisal, not the result.

In the TCTSA-R, we propose a more parsimonious integration of Lazarusian ideas of cognitive appraisal and challenge and threat, and the cognitive appraisal and challenge and threat concepts put forth in the TCTSA. A recent critical review has proposed that challenge and threat states could be simultaneously activated, this co-activation can accordingly lead to individuals appraising motivated performance situations like sport as both a challenge, a threat, both, or neither (Uphill et al., 2019). Although at this time, there is no direct evidence that individuals can be challenged and threatened at the same time, in our revision, we consider that challenge and threat states are not static, and that individuals can move from one state to another. This revision is important, because it reflects more realistically and comprehensively the cognitive operations that take place when an athlete is approaching a motivated performance situation. Specifically, we include primary appraisals according to Lazarus (1999), and detail how an initial challenge appraisal could still lead to poor performance through a perception of low resource appraisals as posited in the TCTSA through reappraisal. Indeed, an athlete can evince a threat state but still perform well so long as they perceive high resources (Turner et al., 2013).

### The Theory of Challenge and Threat States in Athletes-Revised

#### Primary Appraisal

The primary appraisal “motivational relevance” will reflect the extent to which the competition is personally relevant to the athlete’s goals. In addition, the primary appraisal “goal congruence” will reflect the extent to which the conditions are favorable for their success. Challenge results from the appraisal that the competition is highly relevant to the athlete’s goals, and that the conditions are favorable for success. Threat results from the appraisal that the competition is highly relevant to the athlete’s goals, and that the conditions are unfavorable for success. Challenge reflects the perception that the athlete can bring the challenge to fruition. Threat reflects the perception that the athlete cannot ameliorate the threat.

#### Demands Versus Resources

Primary appraisal is not the end of the story. It is possible to make an appraisal of threat, but still perceive that you have more than sufficient resources to meet the perceived demands of the situation, and thus approach competition in a challenge state. Taken from the BPSM, demand appraisals comprise perceptions of danger (physical and esteem), uncertainty, and the requirement of effort (physical and mental). The demand appraisals are distinct from primary appraisals. That is, just because a competition is appraised as personally relevant and incongruent with one’s goals (primary appraisal of threat) does not automatically mean that the competition is also perceived as dangerous, uncertain, and effortful (demand appraisal). In addition, even if the competition is appraised as highly demanding, this does not automatically mean that a threat state will prevail, because the individual may perceive more than sufficient resources to meet the perceived demands. That is, in light of primary appraisal and demand appraisal, an athlete can still believe that they have the skills to succeed (high self-efficacy), that they have control over those skills (high control), and that their social environment is conducive to success (high perceived social support) (i.e., sufficient resource appraisals).

In contrast, it is possible to make a primary appraisal of challenge but also believe that you do not have sufficient resources to meet the perceived demands of the competition, and thus approach the competition in a threat state. That is, an athlete who appraises a competition as personally relevant and *congruent* with one’s goals (primary appraisal of challenge) can also perceive high danger, high uncertainty, and a high requirement for effort, and believe that they do not have the skills to succeed (low self-efficacy), that they do not have control over their skills (low control), and that their social environment is not conducive to success (low perceived social support) (i.e., insufficient resource appraisals). In other words, the extent to which challenge or threat states dominate in anticipation of a competitive situation is dependent on the primary appraisal of challenge and threat, the perceived demands of the competition, and extent to which personal and social resources meet or exceed the demands.

Therefore, the extent to which perceived resources meet or exceed demands could operate as a bifurcation factor that dictates the affective, cardiovascular, and performance outcomes of the competing athlete. That is, in the event of a challenge primary appraisal, high perceived resources compared to demands are likely to help the athlete to fulfill their potential, whereas low perceived recourses compared to demands are less likely to help the athlete to fulfill their potential. Just because the athlete appraises that conditions are favorable for their performance (challenge), their performance is still in part dependent on how their resources compare to the demands of the competition. By perceiving that resources sufficiently meet the demands, the athlete can bring the challenge to fruition and execute their performance within the perceived favorable conditions. If challenge predominates, it is then likely that a challenge CV pattern is evinced, alongside the recruitment of effective attentional and motor skills required for successful skilled performance (fulfilling of potential). By perceiving that resources do not meet the demands, the athlete cannot bring the challenge to fruition and cannot execute their performance within the perceived favorable conditions. As a result, challenge cannot predominate, it is less likely that a challenge CV pattern is evinced, and less likely that effective attentional and motor skills are recruited, thus undermining the athlete’s ability to fulfill their potential.

In the event of a threat primary appraisal, perceiving that resources exceed the demands of the competition could also help the athlete to fulfill their potential, whereas insufficient recourses could significantly harm the athlete’s performance. By perceiving that resources do not sufficiently meet the demands, the athlete cannot ameliorate the threat and cannot execute their performance within the perceived unfavorable conditions. If threat predominates, it is then likely that a threat CV pattern is evinced, alongside ineffective attentional and motor skills recruitment required for successful skilled performance (not fulfilling of potential). By perceiving that resources do meet the demands, the athlete can ameliorate the threat and execute their performance within the perceived unfavorable conditions. As a result, threat cannot predominate, and it is less likely that a threat CV pattern is evinced, and the athlete is more likely to be able to recruit effective attentional and motor skills required for successful skilled performance (fulfilling of potential).

Therefore, given that an athlete can make both challenge and threat primary appraisals, and can have both high or low resources compared to perceived demands, we propose a 2 × 2 bifurcation theory of challenge and threat, which reflects polychotomy of four parts: high challenge, low challenge, low threat, and high threat. Details of each are given below.

#### High Challenge

High challenge would occur in situations where the athlete perceives high motivational relevance (“there is a goal at stake”), high goal congruence (“conditions are favorable for success”) that results in challenge. The athlete perceives sufficient resources to meet perceived demands. Specifically, the athlete perceives high levels of self-efficacy, control, is focused on approach goals rather than avoidance goals, and has a high perception of available social support, and thus believes that they can bring the challenge to fruition. In other words, they believe that they can make the most of the favorable conditions in this important competition. As a result, the athlete is more likely to experience positive emotions; if negative emotions are experienced, they are perceived as facilitative. The athlete also evinces challenge CV reactivity resulting from a quick SNS response which quickly habituates (cf. Epel et al., 2018). Athletes who respond in this state will also have greater levels of NPY and oxytocin. Consequently, the athlete is more likely to experience helpful performance mechanisms and is therefore likely to fulfill their potential in that competition.

#### Low Challenge

Low challenge would occur in situations where the athlete perceives high motivational relevance (“there is a goal at stake”), high goal congruence (“conditions are favorable for success”) that results in challenge. Specifically, the athlete perceives insufficient resources to meet perceived demands. The athlete perceives low levels of self-efficacy, control, is focused on avoidance goals rather than approach goals, and has a low perception of available social support, and thus believes that they cannot bring the challenge to fruition. In other words, they believe that they cannot make the most of the favorable conditions in this important competition. Thus, the situation is perceived as favorable but the personal resources are not. As a result, the athlete is likely to experience positive and negative emotions, but perceives negative emotions as debilitative. The athlete evinces challenge CV reactivity to a lesser extent than when in high challenge. Although the athletes show challenge CV reactivity, the SNS response does not habituate as quickly as under conditions of high challenge. It is also proposed that athletes who respond in this state will also have low levels of NPY and oxytocin reflecting, in part, a low level of resources to meet the demands. Consequently, the athlete is less likely to experience helpful performance mechanisms and is less likely to fulfill their potential in that competition compared to high challenge.

#### High Threat

High threat would occur in situations where the athlete perceives high motivational relevance (“there is a goal at stake”), low goal congruence (“conditions are not favorable for success”) that results in threat. Specifically, the athlete perceives insufficient resources to meet perceived demands. The athlete perceives low levels of self-efficacy, control, is focused on avoidance goals rather than approach goals, and has a low perception of available social support, and thus believes that they cannot ameliorate the threat. In other words, they believe that they cannot overcome the unfavorable conditions in this important competition. As a result, the athlete is likely to experience negative emotions, and perceive negative emotions as debilitative. The athletes evince threat CV reactivity and the SNS response takes longest to habituate. Athletes in this group also have low levels of NPY and oxytocin. Consequently, the athlete is likely to experience unhelpful performance mechanisms (attention etc.) and is unlikely to fulfill their potential in that competition.

#### Low Threat

Low threat would occur in situations where the athlete perceives high motivational relevance (“there is a goal at stake”), low goal congruence (“conditions are not favorable for success”) that results in threat. The athlete perceives sufficient resources to meet perceived demands. Specifically, the athlete perceives high levels of self-efficacy, control, is focused on approach goals rather than avoidance goals, and has a high perception of available social support, and thus believes that they can ameliorate the threat. In other words, they believe that they can overcome the unfavorable conditions in this important competition. As a result, the athlete is likely to experience negative and positive emotions, but perceive negative emotions as facilitative. The athlete evinces less threat CV reactivity than in high threat. Although the athlete evinces threat CV reactivity, the SNS response habituates quicker than in high threat. Athletes in this group will have high levels of NPY and oxytocin, reflecting their perception of sufficient resources to meet the demands. Consequently, the athlete is less likely to experience unhelpful performance mechanisms (such as attention) and is less unlikely to fulfill their potential in that competition.

#### Reappraisal

It is important to clarify where appraisals fit within the TCTSA-R, especially in relation to demand and resource appraisals. In essence, the demand-resource appraisal formula is part of a reappraisal process that takes place iteratively in light of changing contextual and cognitive information that could alter both demand and resource appraisals (Cox, 1978; Lazarus, 1999). In reaction to a primary appraisal of threat for example, athletes appraise the situational demands, and recruit resource appraisals to try to ameliorate this threat, which in effect serves as reappraisal. Thus, primary challenge and threat appraisals do not have to “define” the approach to competition. Essentially, a threat appraisal can be adaptive and welcome, and an athlete can still perform well, so long as they perceive high resources compared to demands. This reappraisal means that individuals can reappraise their initial challenge or threat appraisal, and dictate the resultant approach to the competition as one of four states: high challenge, low challenge, low threat, and high threat.

**Figure 1:**
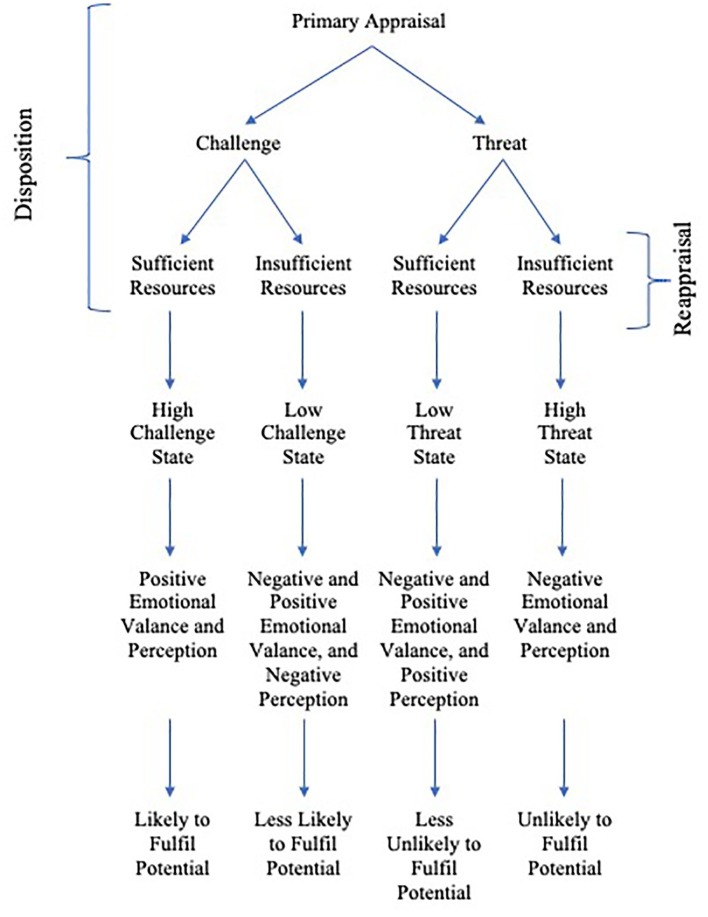
Revised theory of challenge and threat states (TCTSA-R).

In Lazarus’ (1999) cognitive appraisal theory, there is more of an emphasis on secondary appraisals when there is a potential for gain (threat appraisal), leading to either effective coping options (low threat) or no, or ineffective coping options (high threat). There is, however, less emphasis on the challenge appraisal, and it is seemingly assumed that the process “stops” after the initial challenge appraisal where it is appraised that there is a potential for gain or growth. This is also where the TCTSA-R deviates from cognitive appraisal theory, we propose that after an initial challenge appraisal, there is still a possibility for a threat state to dominate, as the resource-demands appraisal can steer challenge and threat states as bifurcation factors (see Figure 1). Thus, we suggest that an athlete can initially appraise a competition as threat, and after reappraising their demands and resources, either challenge or threat dominates, but four states are possible. Similarly, after reappraisal, an initial threat appraisal can lead to challenge or threat states.

## Guidance for Research and Applied Work

Taking into account the revised TCTSA, the next step is to pose suggestions for research ideas and applied implications. With these suggestions, it does need to be considered that the TCTSA is a framework for managing stress (Turner and Jones, 2014), and therefore these suggestions are provided within this realm, focusing on demands and resources.

### Suggestions for Research Directions

We propose four broad suggestions for research moving forward, these are around measurement tools, transparency of reporting the (physiological) data including standardized procedures and reporting for physiological measures of challenge and threat, reconsideration of resources and social support, and consideration of behavioral outcomes such as decision-making.

First, the review of the literature raised questions about the measurement approaches that have been taken when measuring the physiological component of challenge and threat states; it is evident that different approaches were taken, especially when considering the reactivity calculations. In light of this, we encourage researchers to focus on considering the durations and time course of the underpinning physiology when measuring physiological responses. Specifically, researchers should assess blood pressure and hemodynamic measures for at least 3 min in the anticipation phase of studies, following task instructions and any manipulation of challenge and threat. Moreover, we recommend that cardiac output and total peripheral resistance are analyzed separately rather than combined into a single index. We also advocate that researchers are transparent when reporting the physiological data, and to consider that individuals can have blunted responses or are “non-responders,” where participants show minimal reactivity (Wormwood et al., 2019) but may still perceive the situation as a motivated performance situation. Therefore, we urge that researchers are more cautious in their decisions as to who to include in their analysis and not, as well as reporting the means of raw scores for the cardiovascular measures. From reviewing past research, it appears that outliers and non-responders are frequently disregarded from the analysis, which can result in flawed conclusions. This is important because it can affect findings and influences the replicability of research findings (Sherwood et al., 1990; Shapiro et al., 1996). Assessing neuroendocrine markers of challenge and threat states, such as cortisol, and NPY, may support our understanding of psychophysiological mechanisms, as would exploring how parasympathetic nervous system activity can also relate to challenge and threat (Laborde et al., 2015; Uphill et al., 2019). Preliminary evidence suggests that high-frequency heart rate variability can be linked to challenge and threat appraisal; Laborde et al. (2015) identified that, compared to baseline, greater threat responses were associated with a decrease in parasympathetic activity and Thornton et al. (2019) found increased HRV after challenge instructions compared with threat instructions.

Second, the measurement tools used for the demand-resource ratio need consideration. One of the more popular measures is the demand-resource evaluation score (DRES; Tomaka et al., 1993). The DRES uses two items from the cognitive appraisal ratio (Tomaka et al., 1993), where one item assesses demands (“How demanding do you expect the task to be?”) and the other assesses coping resources (“How able are you to cope with the demands of the task?”). Logically, only the second question is valuable since it measures the perception that the individual has the resources to meet the demands, regardless of how high the demands are scored. Other measures that have been used are the recently developed Challenge and Threat in Sport (CAT-Sport) Scale (Rossato et al., 2018), and 11 items (six assessing demands, five assessing resources) developed by Mendes et al. (2007) for experimental work. In addition, studies that more closely aligned with the TCTSA assess the resources *via* separate measures of self-efficacy, perceived control, and goal achievement (i.e., Meijen et al., 2013, 2014; Turner et al., 2013). None of the aforementioned psychometrics measure challenge and threat cognitive appraisals accurately in line with the TCTSA. Therefore, clearly a valuable line of research is to develop such a measure and validity test it across multiple sport participation levels.

Third, the role of social support in appraisal processes has received limited attention. Information about whether a situation is to be perceived as a threat is frequently derived from others (e.g., Maratos, 2011). Moreover, support as a resource might influence appraisal process in varying ways depending on whether it is perceived or received, the type of support offered (e.g., instrumental or emotional), and the source of support. For example, support from a coach might be more potent than that offered from a friend or stranger, at least in some performance situations. There is some evidence that psychological interventions are associated with larger benefits when they are delivered by coaches rather than strangers (Brown and Fletcher, 2017). Whereas there is an extensive literature focusing on social support and cardiovascular reactions to stress (e.g., Teoh and Hilmert, 2018), understanding how social support influences appraisal processes or hemodynamic alterations in anticipation of performance would aid our understanding of challenge and threat states.

Finally, we suggest that future research considers the outcome measures used and re-evaluates the pathways used to measure performance. To date, most of the challenge and threat literature has focused on overall sport performance indices. In only one study (Turner et al., 2012) was decision-making assessed through use of the Stroop task. Other decision-making tasks could be used to assess system 1(automatic and quick) and system 2 (diverting attention to effortful mental activities) processes (Kahneman, 2011). For example, Simonovic et al. (2017) found that stress was associated with poorer Iowa Gambling Task and Cognitive Reflection Task performance. Similarly, only one study has focused on (physical) power (Wood et al., 2018b) as an alternative outcome measure for performance; thus further studies of antecedents of overall sport performance and their relation to challenge and threat states are encouraged.

### Applied Suggestions

The evaluation of the balance between demands and resources is at the core of challenge and threat states, and therefore the guidance for applied work will focus on adjusting the demands and enhancing the resources. As posed in the TCTSA-R, one can still fulfill potential in low challenge appraisal, and in high challenge appraisal you can still fail; therefore, we focus on suggestions to help individuals to develop what it requires to move to a challenge state.

#### Changing Demands

One way of altering the demands is by implementing standardized protocols that are focused on providing instructions that are related to uncertainty, potential for danger, and effort. Studies have demonstrated that using protocols altering the demands of a sporting situation influence challenge and threat states. These instructions have focused on informing athletes that their performance will be compared to others, that they will be evaluated by coaching staff, and that their score is to be taken into account for future team selections (Moore et al., 2012; Turner et al., 2013). Building on this, pressure training (for example, see Stoker et al., 2017) can also be considered as a means to helping athletes reduce the demands of a situation through the process of being more familiar with the situation and thus reducing the uncertainty, potential for danger, and effort required. For example, in one study (Turner et al., 2013), a pressured batting test was developed that emphasized the ego-threatening nature of the task. Elite cricket athletes were instructed that a Batting Test would assess their ability to perform under pressure, that they would be required to face 30 balls and attain 36 runs in order to be successful, and that their total score would be compared to all other participants. The instructions also stated that coaches would consider their performance in the Batting Test when making future decisions about program selection, and therefore they would have to try very hard to perform well. The use of pressure testing like the Batting Test may be a useful way of regularly and systematically introducing athletes to pressure in a training context. Desensitization research suggests that repeated exposure to these types of activities could help athletes to adapt to stressful situations (Wolpe, 1973), thus becoming better prepared for actual competitive pressure (Jones and Turner, 2014).

Altering task instructions can have implications for how coaches communicate with athletes, and coaches can indirectly instigate a threat state when drawing on task instructions that are focused on increasing the demands, but have an athlete who does not perceive to have the resources such as self-efficacy or a sense of perceived control. What should also be considered is that changing the demands is less within a person’s control than enhancing cognitive resources. That is, one may rely on others, such as a coach, to alter the environmental demands. Moreover, despite athletes experiencing a cardiovascular reactivity pattern indicative of a threat, this did not always affect performance, especially when these athletes have higher levels of self-efficacy (Turner et al., 2013). Considering that self-efficacy, together with perceived control and approach/avoidance goals, is a cognitive resource in the TCTSA, we suggest adopting an applied focus that is more within an individual’s control by focusing on resources.

#### Enhancing Resources

To develop cognitive resources such as self-efficacy, perceived control, and emotion control, practical psychological skill interventions can be implemented, where a strategic focus is placed on enhancing self-efficacy, perceived control, and emotion control through the implementation of psychological techniques including imagery, goal-setting, concentration, and self-talk (Andersen, 2009). Findings from challenge and threat research have demonstrated that imagery scripts can differentiate between challenge and threat states (Williams et al., 2010) rather than just focusing on using imagery to manipulate challenge and threat states; this can be built on to strengthen challenge states. Also, based on the emerging evidence that irrational beliefs, as proposed within REBT, are related to greater threat (Dixon et al., 2017; Evans et al., 2018), and that rational self-talk has been shown to increase performance under pressure (Turner et al., 2018), REBT could be applied with athletes in order to promote rational beliefs, and subsequent challenge appraisals. Indeed, the use of REBT in sport is growing (Turner, 2016), with some research finding that systolic blood pressure is reduced in athletes following REBT (Wood et al., 2018a). Future research could examine how REBT can influence challenge and threat states.

## Conclusion

How individuals approach motivated performance situations in a competitive sporting environment has been the focus of many researchers in the field of sport psychology and beyond. Reviewing the research related to challenge and threat states inspired revisions to the Theory of Challenge and Threat States. In particular, we suggest that NPY and oxytocin are also key indicators for facilitating a challenge state. Moreover, we introduced a 2 × 2 bifurcation theory of challenge and threat reflecting the polychotomy of high challenge, low challenge, low threat, and high threat. These revisions to the TCTSA are intended to stimulate more research around measurement tools and reconsideration of resources including social support. Finally, from an applied perspective, the revisions highlight the potential for working toward a challenge state based on adjusting demands and enhancing resources.

## Author Contributions

CM compiled Table 1 and was responsible for the organization of the manuscript. MT designed Figure 1. MT and CM reviewed the challenge and threat research. CM, MT, MJ, DS, and PM contributed to the writing of the manuscript.

### Conflict of Interest

The authors declare that the research was conducted in the absence of any commercial or financial relationships that could be construed as a potential conflict of interest.

## Footnotes

^1^The scenario described in this paragraph is hypothetical and Jessica and Sarah are fictional characters.

## References

[ref1] Allen M. S. Frings D. Hunter S. (2012). Personality, coping, and challenge and threat states in athletes. Int. J. Sport Exerc. Psychol. 10, 264–275. 10.1080/1612197X.2012.682375

[ref2] Andersen M. B. (2009). “The ‘canon’ of psychological skills training for enhancing performance” in Performance psychology in action: A casebook for working with athletes, performing artists, business leaders, and professionals in high-risk occupations. ed. Hays K. F. (Washington, DC: American Psychological Association), 11–34.

[ref3] Antonijevic I. A. Murck H. Bohlhalter S. Frieboes R. M. Holsboer F. Steiger A. (2000). Neuropeptide Y promotes sleep and inhibits ACTH and cortisol release in young men. Neuropharmacology 39, 1474–1481. 10.1016/S0028-3908(00)00057-5, PMID: 10818263

[ref4] Arnold R. Edwards T. Rees T. (2018). Organizational stressors, social support, and implications for subjective performance in high-level sport. Psychol. Sport Exerc. 39, 204–212. 10.1016/j.psychsport.2018.08.010

[ref5] Bandura A. (1997). Self-efficacy: The exercise of control. New York, NY: Freeman.

[ref6] Baumeister R. F. Leary M. R. (1995). The need to belong: desire for interpersonal attachments as a fundamental human motivation. Psychol. Bull. 117, 497–529. 10.1037/0033-2909.117.3.497, PMID: 7777651

[ref7] Behnke M. Kaczmarek L. D. (2018). Successful performance and cardiovascular markers of challenge and threat: a meta-analysis. Int. J. Psychophysiol. 130, 73–79. 10.1016/j.ijpsycho.2018.04.007, PMID: 29680521

[ref8] Bianco T. Eklund R. C. (2001). Conceptual consideration for social support research in sport and exercise settings: the case of sport injury. J. Sport Exerc. Psychol. 23, 85–107. 10.1123/jsep.23.2.85

[ref9] Blascovich J. (2008). “Challenge and threat” in Handbook of approach and avoidance motivation. ed. Elliot A. J. (New York, NY: Psychology Press), 431–445.

[ref10] Blascovich J. Mendes W. B. (2000). “Challenge and threat appraisals: the role of affective cues” in Feeling and thinking: The role of affect in social cognition. ed. Forgas J. P. (Paris: Cambridge University Press), 59–82.

[ref11] Blascovich J. Mendes W. B. Tomaka J. Salomon K. Seery M. (2003). The robust nature of the biopsychosocial model challenge and threat: a reply to Wright and Kirby. Personal. Soc. Psychol. Rev. 7, 234–243. 10.1207/S15327957PSPR0703_03 12788689

[ref12] Blascovich J. Seery M. D. Mugridge C. A. Norris R. K. Weisbuch M. (2004). Predicting athletic performance from cardiovascular indexes of challenge and threat. J. Exp. Soc. Psychol. 40, 683–688. 10.1016/j.jesp.2003.10.007

[ref13] Blascovich J. Tomaka J. (1996). The biopsychosocial model of arousal regulation. Adv. Exp. Soc. Psychol. 28, 1–51.

[ref14] Brimmell J. Parker J. Wilson M. R. Vine S. J. Moore L. J. (2019). Challenge and threat states, performance, and attentional control during a pressurized soccer penalty task. Sport Exerc. Perform. Psychol. 8, 63–79. 10.1037/spy0000147

[ref15] Brown D. J. Fletcher D. (2017). Effects of psychological and psychosocial interventions on sport performance: a meta-analysis. Sports Med. 47, 77–99. 10.1007/s40279-016-0552-7, PMID: 27241124

[ref16] Cardoso C. Ellenbogen M. A. Orlando M. A. Bacon S. L. Joober R. (2013). Intranasal oxytocin attenuates the cortisol response to physical stress: a dose-response study. Psychoneuroendocrinology 38, 399–407. 10.1016/j.psyneuen.2012.07.013, PMID: 22889586

[ref17] Cardoso C. Kingdon D. Ellenbogen M. A. (2014). A meta-analytic review of the impact of intranasal oxytocin administration on cortisol concentrations during laboratory tasks: moderation by method and mental health. Psychoneuroendocrinology 49, 161–170. 10.1016/j.psyneuen.2014.07.014 25086828

[ref18] Carroll D. Sheffield D. (1998). Social psychophysiology, social circumstances, and health. Ann. Behav. Med. 20, 333–337. 10.1007/BF02886383, PMID: 10234428

[ref19] Carter J. R. Goldstein D. S. (2015). Sympathoneural and adrenomedullary responses to mental stress. Compr. Physiol. 5, 119–146. 10.1002/cphy.c140030, PMID: 25589266 PMC5280073

[ref20] Chadha N. J. Turner M. J. Slater M. J. (2019). Investigating irrational beliefs, cognitive appraisals, challenge and threat, and affective states in golfers approaching competitive situations. Front. Psychol. 10:2295. 10.3389/fpsyg.2019.02295, PMID: 31649600 PMC6795749

[ref21] Closa León T. Nouwen A. Sheffield D. (2007). Social support and individual variability in patterns of haemodynamic reactivity and recovery. Psychol. Health 22, 473–492. 10.1080/14768320600941806

[ref22] Cohen S. Gottlieb B. H. Underwood L. G. (2000). “Social relationships and health” in Social support measurement and intervention: A guide for health and social scientists. eds. Cohen S. Underwood L. G. Gottlieb B. H. (New York, NY: Oxford University Press), 1–25.

[ref23] Cox T. (1978). Stress. London: Macmillan Press.

[ref24] Crocker P. R. E. (1992). Managing stress by competitive athletes: ways of coping. Int. J. Sport Psychol. 23, 161–175.

[ref25] Cumming S. J. Turner M. J. Jones M. (2017). Longitudinal changes in elite rowers’ challenge and threat appraisals of pressure situations: a season-long observational study. Sport Psychol. 31, 217–226. 10.1123/tsp.2016-0087

[ref26] David D. Schnur J. Belloiu A. (2002). Another search for the “hot” cognitions: appraisal, irrational beliefs, attributions, and their relation to emotion. J. Ration. Emot. Cogn. Behav. Ther. 20, 93–131. 10.1023/A:1019876601693

[ref27] DeFreese J. D. Smith A. L. (2014). Athlete social support, negative social interactions and psychological health across a competitive sport season. J. Sport Exerc. Psychol. 38, 619–630. 10.1123/jsep.2014-0040 25602144

[ref28] Di Corrado D. Vitali F. Robazza C. Bortoli L. (2015). Self-efficacy, emotional states, and performance in carom billiards. Percept. Mot. Skills 121, 14–25. 10.2466/30.PMS.121c11x6, PMID: 26226286

[ref29] Didymus F. F. Fletcher D. (2017). Effects of a cognitive-behavioral intervention on field hockey players’ appraisals of organizational stressors. Psychol. Sport Exerc. 30, 173–185. 10.1016/j.psychsport.2017.03.005

[ref30] Dienstbier R. A. (1989). Arousal and physiological toughness: implications for mental and physical health. Psychol. Rev. 96, 84–100. 10.1037/0033-295X.96.1.84, PMID: 2538855

[ref31] Ditzen B. Schaer M. Gabriel B. Bodenmann G. Ehlert U. Heinrichs M. (2009). Intranasal oxytocin increases positive communication and reduces cortisol levels during couple conflict. Biol. Psychiatry 65, 728–731. 10.1016/j.biopsych.2008.10.011, PMID: 19027101

[ref32] Dixon M. Jones M. V. Turner M. J. (2019). The benefits of a challenge approach on match day: investigating cardiovascular reactivity in professional academy soccer players. Eur. J. Sport Sci. 10.1080/17461391.2019.1629179, PMID: 31167615

[ref33] Dixon M. Turner M. J. Gillman J. (2017). Examining the relationships between challenge and threat cognitive appraisals and coaching behaviours in football coaches. J. Sports Sci. 35, 2446–2452. 10.1080/02640414.2016.1273538 28019726

[ref34] Elliot A. J. McGregor H. A. (2001). A 2 × 2 achievement goal framework. J. Pers. Soc. Psychol. 80, 501–519. 10.1037/0022-3514.80.3.501, PMID: 11300582

[ref35] Epel E. S. Crosswell A. D. Mayer S. E. Prather A. A. Slavich G. M. Puterman E.. (2018). More than a feeling: a unified view of stress measurement for population science. Front. Neuroendocrinol. 49, 146–169. 10.1016/j.yfrne.2018.03.001, PMID: 29551356 PMC6345505

[ref36] Evans A. L. Turner M. J. Pickering R. Powditch R. (2018). The effects of rational and irrational coach team talks on the cognitive appraisal and achievement goal orientation of varsity football athletes. Int. J. Sports Sci. Coach. 13, 431–438. 10.1177/1747954118771183

[ref37] Eysenck M. W. Derakshan N. Santos R. Calvo M. G. (2007). Anxiety and cognitive performance: attentional control theory. Emotion 7, 336–353. 10.1037/1528-3542.7.2.336, PMID: 17516812

[ref38] Freeman P. Rees T. (2009). How does perceived support lead to better performance? An examination of potential mechanisms. J. Appl. Sport Psychol. 21, 429–441. 10.1080/10413200903222913

[ref39] Freeman P. Rees T. (2010). Perceived social support from team-mates: direct and stress buffering effects on self-confidence. Eur. J. Sport Sci. 10, 59–67. 10.1080/17461390903049998

[ref40] Frings D. Rycroft N. Allen M. S. Fenn R. (2014). Watching for gains and losses: the effects of motivational challenge and threat on attention allocation during a visual search task. Motiv. Emot. 38, 513–522. 10.1007/s11031-014-9399-0

[ref41] Gaab J. Rohleder N. Nater U. M. Ehlert U. (2005). Psychological determinants of the cortisol stress response: the role of anticipatory cognitive appraisals. Psychoneuroendocrinology 30, 599–610. 10.1016/j.psyneuen.2005.02.001, PMID: 15808930

[ref42] Gramer M. Reitbauer C. (2010). The influence of social support on cardiovascular responses during stressor anticipation and active coping. Biol. Psychol. 85, 268–274. 10.1016/j.biopsycho.2010.07.013, PMID: 20673842

[ref43] Gross J. J. (1998). The emerging field of emotion regulation: an integrative review. Rev. Gen. Psychol. 2, 271–299. 10.1037/1089-2680.2.3.271

[ref44] Hase A. O’Brien J. Moore L. J. Freeman P. (2019). The relationship between challenge and threat states and performance: a systematic review. Sport Exerc. Perform. Psychol. 8, 123–144. 10.1037/spy0000132

[ref45] Heinrichs M. Baumgartner T. Kirschbaum C. Ehlert U. (2003). Social support and oxytocin interact to suppress cortisol and subjective responses to psychosocial stress. Biol. Psychiatry 54, 1389–1398. 10.1016/S0006-3223(03)00465-7, PMID: 14675803

[ref46] Herman J. P. McKlveen J. M. Ghosal S. Kopp B. Wulsin A. Makinson R. (2016). Regulation of the hypothalamic-pituitary-adrenocortical stress response. Compr. Physiol. 6, 603–621. 10.1002/cphy.c150015 27065163 PMC4867107

[ref47] Howle T. C. Eklund R. C. (2013). The effect of induced self-presentation concerns on cognitive appraisal and affect. Anxiety Stress Coping 26, 700–710. 10.1080/10615806.2013.763934, PMID: 23351127

[ref48] Huber M. E. Brown A. J. Sternad D. (2016). Girls can play ball: stereotype threat reduces variability in a motor skill. Acta Psychol. 169, 79–87. 10.1016/j.actpsy.2016.05.010, PMID: 27249638 PMC4987161

[ref49] Jamieson J. P. Mendes W. B. Blackstock E. Schmader T. (2010). Turning the knots in our stomach into bows: reappraisal arousal improves performance on the GRE. J. Exp. Soc. Psychol. 46, 208–212. 10.1016/j.jesp.2009.08.015, PMID: 20161454 PMC2790291

[ref50] Jamieson J. P. Nock M. K. Mendes W. B. (2013). Changing the conceptualization of stress in social anxiety disorder: affective and physiological consequences. Clin. Psychol. Sci. 1, 363–374. 10.1177/2167702613482119

[ref51] Jones G. (1995). More than just a game: research developments and issues in competitive anxiety in sport. Br. J. Psychol. 86, 449–478. 10.1111/j.2044-8295.1995.tb02565.x, PMID: 8542197

[ref52] Jones M. V. Meijen C. McCarthy P. J. Sheffield D. (2009). A theory of challenge and threat states in athletes. Int. Rev. Sport Exerc. Psychol. 2, 161–180. 10.1080/17509840902829331 PMC7016194 32116930

[ref53] Jones M. V. Turner M. J. (2014). “Self-regulation” in Encyclopedia of sport and exercise psychology. eds. Eklund R. C. Tenenbaum G. (Thousand Oaks, CA: Sage Publications).

[ref54] Jones H. S. Williams E. L. Marchant D. Sparks S. A. Bridge C. A. Midgley A. W.. (2016). Improvements in cycling time trial performance are not sustained following the acute provision of challenging and deceptive feedback. Front. Physiol. 7:399. 10.3389/fphys.2016.00399, PMID: 27713701 PMC5031686

[ref55] Kahneman D. (2011). Thinking, fast and slow. New York, NY: Farrar, Straus and Giroux.

[ref56] Kingsbury A. Gaudreau P. Hill K. Coplan R. J. (2014). The influence of social evaluative threat on the putting stroke in golf. Int. J. Golf Sci. 2, 176–194. 10.1123/ijgs.2014-0007

[ref57] Kirsch J. A. Lehman B. J. (2015). Comparing visible and invisible social support: non-evaluative support buffers cardiovascular responses to stress. Stress. Health 31, 351–364. 10.1002/smi.2558, PMID: 24449558

[ref58] Kirschbaum C. Hellhammer D. H. (1994). Salivary cortisol in psychoneuroendocrine research: recent developments and applications. Psychoneuroendocrinology 19, 313–333. 10.1016/0306-4530(94)90013-2, PMID: 8047637

[ref59] Kubzansky L. D. Mendes W. B. Appleton A. A. Block J. Adler G. K. (2012). A heartfelt response: oxytocin effects on response to social stress in men and women. Biol. Psychol. 90, 1–9. 10.1016/j.biopsycho.2012.02.010, PMID: 22387929 PMC3327158

[ref60] Laborde S. Lautenbach F. Allen M. S. (2015). The contribution of coping-related variables and heart rate variability to visual search performance under pressure. Physiol. Behav. 139, 532–540. 10.1016/j.physbeh.2014.12.003, PMID: 25481358

[ref61] Lazarus R. S. (1999). Stress and emotion: A new synthesis. New York: Springer Publishing Company.

[ref62] Lazarus R. S. Folkman S. (1984). Stress, appraisal, and coping. New York: Springer.

[ref63] Linnen A.-M. Ellenbogen M. A. Cardoso C. Joober R. (2012). Intranasal oxytocin and salivary cortisol concentrations during social rejection in university students. Stress 15, 393–402. 10.3109/10253890.2011.631154 22044077

[ref64] Madden C. C. Kirkby R. J. McDonald D. (1989). Coping styles of competitive middle distance runners. Int. J. Sport Psychol. 20, 287–296.

[ref65] Maratos F. A. (2011). Temporal processing of emotional stimuli: the capture and release of attention by angry faces. Emotion 11, 1242–1247. 10.1037/a0024279, PMID: 21942702

[ref66] Massey W. V. Meyer B. B. Naylor A. H. (2013). Toward a grounded theory of self-regulation in mixed martial arts. Psychol. Sport Exerc. 14, 12–20. 10.1016/j.psychsport.2012.06.008

[ref68] Meijen C. Jones M. V. McCarthy P. J. Sheffield D. Allen M. S. (2013). Cognitive and affective components of challenge and threat states. J. Sports Sci. 31, 847–855. 10.1080/02640414.2012.753157, PMID: 23256682

[ref69] Meijen C. Jones M. V. Sheffield D. McCarthy P. J. (2014). Challenge and threat states: cardiovascular, affective, and cognitive responses to a sports-related speech task. Motiv. Emot. 38, 252–262. 10.1007/s11031-013-9370-5

[ref70] Mendes W. B. Gray H. M. Mendoza-Denton R. Major B. Epel E. S. (2007). Why egalitarianism might be good for your health: physiological thriving during stressful intergroup encounters. Psychol. Sci. 18, 991–998. 10.1111/j.1467-9280.2007.02014.x 17958714 PMC2430625

[ref71] Moore L. J. Vine S. J. Freeman P. Wilson M. R. (2013a). Quiet eye training promotes challenge appraisals and aids performance under elevated anxiety. Int. J. Sport Exerc. Psychol. 11, 169–183. 10.1080/1612197X.2013.773688

[ref72] Moore L. J. Vine S. J. Wilson M. R. Freeman P. (2012). The effect of challenge and threat states on performance: an examination of potential mechanisms. Psychophysiology 49, 1417–1425. 10.1111/j.1469-8986.2012.01449.x, PMID: 22913339 PMC3677799

[ref73] Moore L. J. Vine S. J. Wilson M. R. Freeman P. (2014). Examining the antecedents of challenge and threat states: the influence of perceived required effort and support availability. Int. J. Psychophysiol. 93, 267–273. 10.1016/j.ijpsycho.2014.05.009, PMID: 24867434

[ref74] Moore L. J. Vine S. J. Wilson M. R. Freeman P. (2015). Reappraising threat: how to optimize performance under pressure. J. Sport Exerc. Psychol. 37, 339–343. 10.1123/jsep.2014-0186, PMID: 26265345

[ref75] Moore L. J. Wilson M. R. Vine S. J. Coussens A. H. Freeman P. (2013b). Champ or chump? Challenge and threat states during pressurized competition. J. Sport Exerc. Psychol. 35, 551–562. 10.1123/jsep.35.6.551, PMID: 24334317

[ref76] Moore L. J. Young T. Freeman P. Sarkar M. (2018). Adverse life events, cardiovascular responses, and sports performance under pressure. Scand. J. Med. Sci. Sports 28, 340–347. 10.1111/sms.12928 28581687

[ref77] Morgan C. A. III Rasmusson A. M. Wang S. Hoyt G. Hauger R. L. Hazlett G. (2002). Neuropeptide-Y, cortisol, and subjective distress in humans exposed to acute stress: replication and extension of previous report. Biol. Psychiatry 52, 136–142. 10.1016/S0006-3223(02)01319-7, PMID: 12114005

[ref78] Morgan C. A. III Wang S. Rassmuson A. Hazlett G. Anderson G. Charney D. S. (2001). Relationship among cortisol, catecholamines, neuropeptide-Y and human performance during exposure to uncontrollable stress. Psychosom. Med. 63, 412–422. 10.1097/00006842-200105000-00010, PMID: 11382268

[ref79] Morgan C. A. III Wang S. Southwick S. M. Rasmusson A. Hauger R. Charney D. S. (2000). Plasma neuropeptide-Y in humans exposed to acute uncontrollable stress. Biol. Psychiatry 47, 902–909. 10.1016/S0006-3223(99)00239-5, PMID: 10807963

[ref80] Mosley E. Laborde S. Kavanagh E. (2017). The contribution of coping related variables and cardiac vagal activity on the performance of a dart throwing task under pressure. Physiol. Behav. 179, 116–125. 10.1016/j.physbeh.2017.05.030, PMID: 28577887

[ref81] Nicholls A. R. Polman R. C. J. Levy A. R. (2012). A path analysis of stress appraisals, emotions, coping, and performance satisfaction among athletes. Psychol. Sport Exerc. 13, 263–270. 10.1016/j.psychsport.2011.12.003

[ref82] Nulk M. Schuh W. Burrell L. M. Matthews M. D. (2011). Effects of neuropeptide Y on resilience to PTSD. West Point Resilience Project (WPRP), Research Report PL488E3. Department of Behavioral Sciences and Leadership.

[ref83] O’Donovan A. Hughes B. M. (2008). Factors that moderate the effect of laboratory-based social support on cardiovascular reactivity to stress. Int. J. Psychol. Psychol. Ther. 8, 85–102.

[ref84] Rees T. I. M. Freeman P. (2009). Social support moderates the relationship between stressors and task performance through self-efficacy. J. Soc. Clin. Psychol. 28, 244–263. 10.1521/jscp.2009.28.2.244

[ref85] Rees T. Hardy L. (2000). An investigation of the social support experiences of high-level sports performers. Sport Psychol. 14, 327–347. 10.1123/tsp.14.4.327

[ref86] Rees T. Hardy L. (2004). Matching social support with stressors: effects on factors underlying performance in tennis. Psychol. Sport Exerc. 5, 319–337. 10.1016/S1469-0292(03)00018-9

[ref87] Rees T. Hardy L. Freeman P. (2007). Stressors, social support, and effects upon performance in golf. J. Sports Sci. 25, 33–42. 10.1080/02640410600702974, PMID: 17127579

[ref88] Rees T. Ingledew D. K. Hardy L. (1996). Dimensions of performance and differential effects of hassles, support and perceived control. J. Sports Sci. 14, 43–44.

[ref89] Robazza C. Izzicupo P. D’Amico M. A. Ghinassi B. Crippa M. C. Di Cecco V.. (2018). Psychophysiological responses of junior orienteers under competitive pressure. PLoS One 13:e0196273. 10.1371/journal.pone.0196273, PMID: 29698498 PMC5919653

[ref90] Robyn J. McQuaid R. A. McInnis O. A. Paric A. Al-Yawer F. Matheson K. (2016). Relations between plasma oxytocin and cortisol: the stress buffering role of social support. Neurobiol. Stress 3, 52–60. 10.1016/j.ynstr.2016.01.001 27981177 PMC5146198

[ref91] Rossato C. J. L. Uphill M. A. Swain J. Coleman D. A. (2018). The development and preliminary validation of the challenge and threat in sport (CAT-sport) scale. Int. J. Sport Exerc. Psychol. 16, 164–177. 10.1080/1612197X.2016.1182571

[ref92] Sammy N. Anstiss P. A. Moore L. J. Freeman P. Wilson M. R. Vine S. J. (2017). The effects of arousal reappraisal on stress responses, performance and attention. Anxiety Stress Coping 30, 619–629. 10.1080/10615806.2017.1330952, PMID: 28535726

[ref93] Sanderson J. (2016). Elite quarterbacks do not laugh when they are losing: exploring fan responses to athletes’ emotional displays. Int. J. Sport Exerc. Psychol. 14, 281–294. 10.1080/1612197X.2015.1023211

[ref94] Seery M. D. (2011). Challenge or threat? Cardiovascular indexes of resilience and vulnerability to potential stress in humans. Neurosci. Biobehav. Rev. 35, 1603–1610. 10.1016/j.neubiorev.2011.03.003, PMID: 21396399

[ref95] Seery M. D. (2013). The biopsychosocial model of challenge and threat: using the heart to measure the mind. Soc. Personal. Psychol. Compass 7, 637–653. 10.1111/spc3.12052

[ref96] Selye H. (1956). The stress of life. New York: McGraw-Hill.

[ref97] Shapiro D. Jamner L. D. Lane J. D. Light K. C. Myrtek M. Sawada Y.. (1996). Blood pressure publication guidelines. Psychophysiology 33, 1–12. 10.1111/j.1469-8986.1996.tb02103.x, PMID: 8570790

[ref98] Sherwood A. Allen M. T. Fahrenberg J. Kelsey R. M. Lovallo W. R. Van Doornen L. J. (1990). Methodological guidelines for impedance cardiography. Psychophysiology 27, 1–23. 10.1111/j.1469-8986.1990.tb02171.x, PMID: 2187214

[ref99] Shumaker S. A. Brownell A. (1984). Toward a theory of social support: closing conceptual gaps. J. Soc. Issues 40, 11–36. 10.1111/j.1540-4560.1984.tb01105.x

[ref100] Simonovic B. Stupple E. J. Gale M. Sheffield D. (2017). Stress and risky decision making: cognitive reflection, emotional learning or both. J. Behav. Decis. Mak. 30, 658–665. 10.1002/bdm.1980

[ref101] Skinner N. Brewer N. (2002). The dynamics of threat and challenge appraisals prior to stressful achievement events. J. Pers. Soc. Psychol. 83, 678–692. 10.1037/0022-3514.83.3.678, PMID: 12219862

[ref102] Slater M. J. Evans A. L. Turner M. J. (2016). Implementing a social identity approach for effective change management. J. Chang. Manag. 16, 18–37. 10.1080/14697017.2015.1103774

[ref103] Slater M. J. Turner M. J. Evans A. L. Jones M. V. (2018). Capturing hearts and minds: the influence of relational identification with the leader on followers’ mobilization and cardiovascular reactivity. Leadersh. Q. 29, 379–388. 10.1016/j.leaqua.2017.08.003

[ref104] Speisman J. C. Lazarus R. S. Mordkoff A. Davison L. (1964). Experimental reduction of stress based on ego-defense theory. J. Abnorm. Soc. Psychol. 68, 367–380. 10.1037/h0048936, PMID: 14136776

[ref105] Stoker M. Maynard I. Butt J. Hays K. Lindsay P. Norenberg D. A. (2017). The effect of manipulating training demands and consequences on experiences of pressure in elite netball. J. Appl. Sport Psychol. 29, 434–448. 10.1080/10413200.2017.1298166

[ref106] Tamminen K. A. Sabiston C. M. Crocker P. R. (2019). Perceived esteem support predicts competition appraisals and performance satisfaction among varsity athletes: a test of organizational stressors as moderators. J. Appl. Sport Psychol. 31, 27–46. 10.1080/10413200.2018.1468363

[ref107] Teoh A. N. Hilmert C. (2018). Social support as a comfort or an encouragement: a systematic review on the contrasting effects of social support on cardiovascular reactivity. Br. J. Health Psychol. 23, 1040–1065. 10.1111/bjhp.12337, PMID: 30084181

[ref108] Thornton C. Sheffield D. Baird A. (2019). Motor performance during experimental pain: the influence of exposure to contact sports. Eur. J. Pain 23, 1020–1030. 10.1002/ejp.1370, PMID: 30697875

[ref109] Tomaka J. Blascovich J. Kelsey R. M. Leitten C. L. (1993). Subjective, physiological, and behavioral effects of threat and challenge appraisal. J. Pers. Soc. Psychol. 65, 248–260. 10.1037/0022-3514.65.2.248

[ref110] Trotman G. P. Williams S. E. Quinton M. L. Veldhuijzen van Zanten J. J. C. S. (2018). Challenge and threat states: examining cardiovascular, cognitive and affective responses to two distinct laboratory stress tasks. Int. J. Psychophysiol. 126, 42–51. 10.1016/j.ijpsycho.2018.02.004, PMID: 29477547

[ref111] Turner M. J. (2016). Rational emotive behavior therapy (REBT), irrational and rational beliefs, and the mental health of athletes. Front. Psychol. 7:1423. 10.3389/fpsyg.2016.01423, PMID: 27703441 PMC5028385

[ref112] Turner M. J. Barker J. B. (2014a). What business can learn from sport psychology. UK: Bennion Kearny.

[ref113] Turner M. J. Barker J. B. (2014b). Tipping the balance: The mental skills handbook for athletes. UK: Bennion Kearny.

[ref114] Turner M. J. Jones M. V. (2014). “Stress, emotions and athletes’ positive adaptation to sport: contributes from a transactional perspective” in Positive human functioning from a multidimensional perspective. Vol. 1 eds. Gomes A. R. Resende R. Albuquerque A. (New York, NY: Nova Science), 85–111.

[ref115] Turner M. J. Jones M. V. Sheffield D. Barker J. B. Coffee P. (2014). Manipulating cardiovascular indices of challenge and threat using resource appraisals. Int. J. Psychophysiol. 94, 9–18. 10.1016/j.ijpsycho.2014.07.004, PMID: 25036595

[ref116] Turner M. J. Jones M. V. Sheffield D. Cross S. L. (2012). Cardiovascular indices of challenge and threat states predict competitive performance. Int. J. Psychophysiol. 86, 48–57. 10.1016/j.ijpsycho.2012.08.004, PMID: 22918086

[ref117] Turner M. J. Jones M. V. Sheffield D. Slater M. J. Barker J. B. Bell J. J. (2013). Who thrives under pressure? Predicting the performance of elite academy cricketers using the cardiovascular indicators of challenge and threat states. J. Sport Exerc. Psychol. 35, 387–397. 10.1123/jsep.35.4.387, PMID: 23966448

[ref118] Turner M. J. Kirkham L. Wood A. G. (2018). Teeing up for success: the effects of rational and irrational self-talk on the putting performance of amateur golfers. Psychol. Sport Exerc. 38, 148–153. 10.1016/j.psychsport.2018.06.012

[ref119] Uchino B. N. Carlisle M. Birmingham W. Vaughn A. A. (2011). Social support and the reactivity hypothesis: conceptual issues in examining the efficacy of received support during acute psychological stress. Biol. Psychol. 86, 137–142. 10.1016/j.biopsycho.2010.04.003, PMID: 20398724 PMC2928410

[ref120] Udry E. (1996). Social support: exploring its role in the context of athletic injuries. J. Sport Rehabil. 5, 151–163. 10.1123/jsr.5.2.151

[ref121] Uphill M. A. Rossato C. Swain J. O’Driscoll J. M. (2019). Challenge and threat: a critical review of the literature and an alternative conceptualisation. Front. Psychol. 10:1255. 10.3389/fpsyg.2019.01255 31312151 PMC6614335

[ref122] Vine S. J. Freeman P. Moore L. J. Chandra-Ramanan R. Wilson M. R. (2013). Evaluating stress as a challenge is associated with superior attentional control and motor skill performance: testing the predictions of the biopsychosocial model of challenge and threat. J. Exp. Psychol. Appl. 19, 185–194. 10.1037/a0034106, PMID: 24059821

[ref123] Vine S. J. Uiga L. Lavric A. Moore L. J. Tsaneva-Atanasova K. Wilson M. R. (2015). Individual reactions to stress predict performance during a critical aviation incident. Anxiety Stress Coping 28, 467–477. 10.1080/10615806.2014.986722, PMID: 25396282

[ref124] Webb R. C. (2003). Smooth muscle contraction and relaxation. Adv. Physiol. Educ. 27, 201–206. 10.1152/advances.2003.27.4.201, PMID: 14627618

[ref125] Williams S. E. Cumming J. Balanos G. M. (2010). The use of imagery to manipulate challenge and threat appraisal states in athletes. J. Sport Exerc. Psychol. 32, 339–358. 10.1123/jsep.32.3.339, PMID: 20587822

[ref126] Wolpe J. (1973). The practice of behavior therapy. New York: Pergamon Press.

[ref127] Wood A. G. Barker J. B. Turner M. J. Sheffield D. (2018a). Examining the effects of rational emotive behavior therapy on performance outcomes in elite paralympic athletes. Scand. J. Med. Sci. Sports 28, 329–339. 10.1111/sms.12926, PMID: 28581692

[ref128] Wood N. Parker J. Freeman P. Black M. Moore L. (2018b). The relationship between challenge and threat states and anaerobic power, core affect, perceived exertion, and self-focused attention during a competitive sprint cycling task. Prog. Brain Res. 240, 1–17. 10.1016/bs.pbr.2018.08.006 30390825

[ref129] Wormwood J. B. Khan Z. Siegel E. Lynn S. K. Dy J. Barrett L. F.. (2019). Physiological indices of challenge and threat: a data-driven investigation of autonomic nervous system reactivity during an active coping stressor task. Psychophysiology 56:e13454. 10.1111/psyp.13454, PMID: 31407813 PMC6803040

[ref130] Wright R. A. Kirby L. D. (2003). Cardiovascular correlates of challenge and threat appraisals: a critical examination of the biopsychosocial analysis. Personal. Soc. Psychol. Rev. 7, 216–233. 10.1207/S15327957PSPR0703_02 12788688

